# Genomic and oncogenic preference of HBV integration in hepatocellular carcinoma

**DOI:** 10.1038/ncomms12992

**Published:** 2016-10-05

**Authors:** Ling-Hao Zhao, Xiao Liu, He-Xin Yan, Wei-Yang Li, Xi Zeng, Yuan Yang, Jie Zhao, Shi-Ping Liu, Xue-Han Zhuang, Chuan Lin, Chen-Jie Qin, Yi Zhao, Ze-Ya Pan, Gang Huang, Hui Liu, Jin Zhang, Ruo-Yu Wang, Yun Yang, Wen Wen, Gui-Shuai Lv, Hui-Lu Zhang, Han Wu, Shuai Huang, Ming-Da Wang, Liang Tang, Hong-Zhi Cao, Ling Wang, Tin-Lap Lee, Hui Jiang, Ye-Xiong Tan, Sheng-Xian Yuan, Guo-Jun Hou, Qi-Fei Tao, Qin-Guo Xu, Xiu-Qing Zhang, Meng-Chao Wu, Xun Xu, Jun Wang, Huan-Ming Yang, Wei-Ping Zhou, Hong-Yang Wang

**Affiliations:** 1 Eastern Hepatobiliary Surgery Hospital, Second Military Medical University, Shanghai 200438, China; 2 National Center for Liver Cancer, Shanghai 200438, China; 3 BGI-Shenzhen, Shenzhen 518083, China; 4 School of Bioscience and Bioengineering, South China University of Technology, Guangzhou 510006, China; 5 Faculty of Medicine, School of Biomedical Sciences, The Chinese University of Hong Kong, Shatin, Hong Kong 999077, China; 6 Department of Vascular and Endocrine Surgery, Xijing Hospital, Fourth Military Medical University, Xi An 710032, China; 7 Department of Biology, University of Copenhagen, Ole Maaløes Vej 5,2200 Copenhagen, Denmark; 8 State Key Laboratory of Oncogenes and Related Genes, Cancer Institute of Renji Hospital, Shanghai Jiaotong University, Shanghai 200127, China

## Abstract

Hepatitis B virus (HBV) can integrate into the human genome, contributing to genomic instability and hepatocarcinogenesis. Here by conducting high-throughput viral integration detection and RNA sequencing, we identify 4,225 HBV integration events in tumour and adjacent non-tumour samples from 426 patients with HCC. We show that HBV is prone to integrate into rare fragile sites and functional genomic regions including CpG islands. We observe a distinct pattern in the preferential sites of HBV integration between tumour and non-tumour tissues. HBV insertional sites are significantly enriched in the proximity of telomeres in tumours. Recurrent HBV target genes are identified with few that overlap. The overall HBV integration frequency is much higher in tumour genomes of males than in females, with a significant enrichment of integration into chromosome 17. Furthermore, a cirrhosis-dependent HBV integration pattern is observed, affecting distinct targeted genes. Our data suggest that HBV integration has a high potential to drive oncogenic transformation.

## Abstract (web summary)

Hepatitis B infection is a risk factor for hepatocellular carcinoma. Here, the authors characterise viral infection in a cohort of hepatocellular carcinoma patients and find viral integration is more frequent in males than females.

## Graphical abstract

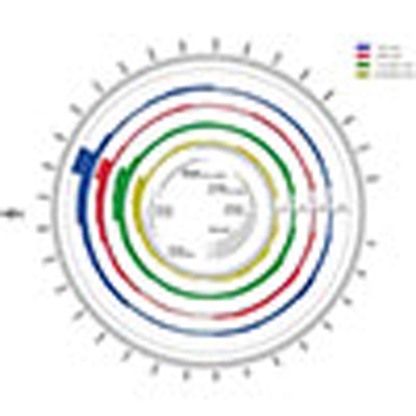

Hepatocellular carcinoma (HCC) ranks fifth in global cancer incidence and represents the third leading cause of cancer deaths^1^. Chronic infection with hepatitis B or C virus (HBV or HCV) represents the major risk factors for the development of HCC^2^,^3^. Unlike HCV, an RNA virus which never integrates into the host genome during its lifecycle, HBV–DNA frequently integrates into host genome and progressively contributes to hepatocarcinogenesis^4^. Integration of HBV DNA into the host genome causes genetic damage and chromosomal instability, which is known to be selectively advantageous for tumour progression^5^. The deregulated host gene expression or a subset of rearranged integration sites has been shown to play a key role in HCC development^6^. In addition, expression of viral proteins such as X protein and S antigen as well as their oncogenic mutants may further enable the acquisition of neoplastic characteristics. Previous isolation of HBV integration sites using PCR-based methods^7^ and more recent use of deep sequencing in a small size cohort of HCC patients suggests that the HBV insertional sites occurred randomly throughout the genome, leading to the presumption that there were no preferential sites of integration. However, recurrent HBV integrations have recently been identified in a large cohort of HCC patients through the application of high-throughput next-generation sequencing^8^,^9^,^10^,^11^. Although recurrent integrations still represented a minority of the total events characterized, the occurrence of recurrent sites of insertion suggests that HBV may have preferential integration sites associated with distinct biological consequences and clinical outcomes. The most common HBV integration event is located at the telomerase reverse transcriptase gene (*TERT*), which is thought to confer early clonal advantage during chronic HBV infection. Other frequently targeted genes such as *KMT2B* and *CCNE1* have recently been identified as new classes of genes not previously known to play a causal role in cancer^10^. However, it is of note that recurrent HBV integration sites identified in previous studies may only represent a minority of recurrent events.

HCC has a male predominance and is closely related to cirrhosis^12^,^13^,^14^. Males with chronic HBV infection are at higher risk of developing HCC compared with females. In fact, the sex difference becomes apparent from the stage of early chronic infection, where the male-to-female ratio increased from 1.2 in asymptomatic carriers to 6.3 in chronic hepatitis and 9.8 in HCC in a Taiwanese study^15^,^16^. On the other hand, although the majority of HCC patients with HBV infection have concurrent liver cirrhosis, the integration of the viral genome into infected cells can directly induce a non-cirrhotic liver to develop HCC. Despite extensive research, the precise mechanisms whereby HBV integration contributes to cirrhotic and non-cirrhotic hepatocarcinogenesis as well as gender disparity remain largely unclear due to technical limitations and a lack of clinical annotation.

In this study, we conducted high-throughput viral integration detection (HIVID), a highly sensitive method for assaying viral insertion compared with the whole-genome sequencing, and analysed 426 HBV-associated HCC patients with or without cirrhosis^17^. Our results revealed an excessive HBV integration across the host genome with particular genomic pattern in a gender or cirrhosis-dependent manner.

## Results

### Characteristics of the HBV integration events in human genome

**Figure 1:**
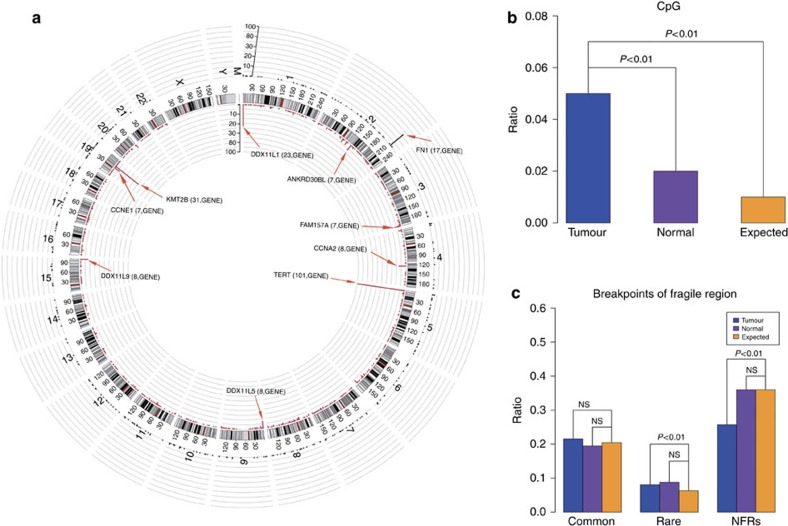
Distribution of HBV integration breakpoints throughout the human genomes in 426 paired samples. (**a**) Distribution of integration breakpoints across the human genome in 426 paired samples. Each bar represents the sample frequency of HBV integration breakpoints at a particular locus in the human genome (hg19). Tumour (red) and non-tumour (dark blue) samples with HBV integrations are shown on the inner and outer circles, respectively. Histogram axis units represent number of samples. Some loci with a high frequency of integration are marked. GENE. (**b**) Comparison of the breakpoints in the CpG island region of 426 paired samples. The expected (assuming uniform, random distribution, yellow) and the observed (actual numbers, tumour: blue; normal liver tissue: purple) percentages of HBV integration breakpoints of tumour and non-tumour samples in CpG islands region are shown. *P* values were calculated by *χ*^2^ test. (**c**) Distribution of integration breakpoints in the fragile region (FR) of 426 paired tumor and non-tumor tissues. The expected (assuming uniform, random distribution, yellow) and the observed (actual numbers, tumour: blue; normal liver tissue: purple) ratios of HBV integration breakpoints in common fragile region, rare fragile region and non-fragile region are shown. *P* values were calculated by *χ*^2^ test. Common: common FR; rare: rare FR; NFRs: non-fragile regions.

To search for HBV integration sites across the HCC genome and decode viral–host interactions, we conducted HIVID analysis on tumour and adjacent non-tumour liver genomes extracted in a cohort including 426 clinically and pathologically well-characterized HBV-associated HCC cases: 298 with cirrhosis and 128 without cirrhosis; 360 males and 66 females (Supplementary Figs 1–3 and Supplementary Table 1). HBV integrations were effectively detected at a single base pair resolution using HBV capture sequencing. A total of 4,225 HBV integration breakpoints were discovered. To confirm the newly discovered events, we randomly selected 180 putative insertions for PCR analysis and successfully validate 81.1% of these integration sites (Supplementary Data 1). Despite a handful of integration hotspots, most insertion sites are distributed in an apparently random manner throughout the genome (Fig. 1a). The integrations maps generated reveal a heterogeneous, widespread viral integration landscape in tumour as well as in non-tumour liver tissue from HCC patients. However, HCC tumour samples and their adjacent non-tumour liver tissues exhibit strikingly distinct patterns of viral insertion. Following the known association between HBV infection and HCC development, tumour samples harbour a much higher frequency of integrated reads than the non-tumour samples. The total HBV integration breakpoints in tumour and non-tumour samples are 3,486 and 739 events, respectively (Supplementary Data 2 and 3). Of 426 paired samples, 76.9% tumour and 37.6% non-tumour tissues contained HBV integration. The average number of integration sites in tumour tissues and adjacent non-tumour liver tissues are 10.6 and 4.6, respectively, (P=3.3 × 10^−6^, unpaired Student's *t* test; Supplementary Fig. 4). Analysis of the distribution of hotspots across the human genome further confirms the prevalence of HBV integrations in tumours. Among the 826 and 303 genes with HBV insertions (intragenic region and upstream 10 kb) detected in tumour and non-tumour tissues, respectively, only 64 genes were shared by the tumour and non-tumour samples, underscoring that integration patterns are distinct in the tumour and normal samples (Fig. 1a and Supplementary Data 4).

### Correlation between HBV integration and genome instability

**Figure 2:**
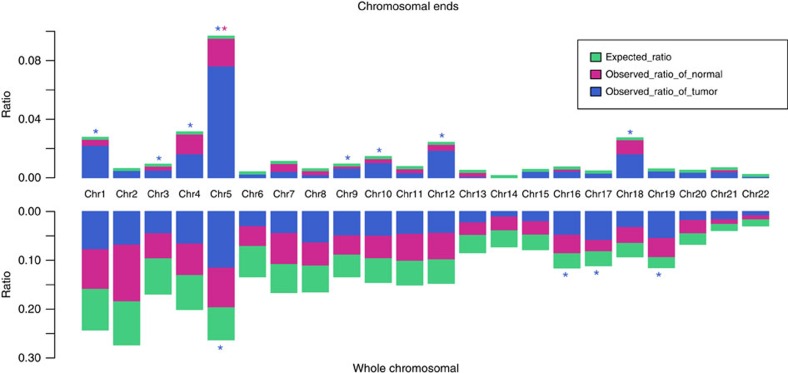
Chromosome enrichment and chromosomal ends enrichment of HBV integration in human genome in 426 paired samples. Each bar of whole-chromosome represents the expected (assuming uniform, random distribution, green) and the observed (actual numbers, tumour: blue, normal liver tissue: purple) ratio of HBV integration breakpoints at a particular chromosome in human genome. Ratios are numbered. Each bar of chromosomal ends represents the expected (assuming uniform, random distribution, green) and the observed (actual numbers, tumor: blue, normal liver tissue, purple) ratio of HBV integration breakpoints at the 2 M region of chromosomal ends in human genome. Ratios are numbered. Dark red star represents statistically significant difference between normal liver samples and random distribution. Blue star represents statistically significant difference between tumour samples and random distribution. *P* values were calculated by *χ*^2^ test.

Statistically significant enrichment of integration was observed within CpG islands in tumours compared with normal samples (Fig. 1b). The integration frequency dramatically decreased in genomic loci moving away from CpG islands. However, no apparent prevalence in integration was observed in transcription factor binding site and transcription start site (Supplementary Fig. 5). HBV was also prone to integrate into rare fragile sites, but only in a tumour-specific context (Fig. 1c). These results suggest preferential HBV integration into chromosomal repetitive or fragile regions could provide a selective advantage during tumorigenesis, rather than representing by product event from random insertion. More importantly, HBV integration sites were significantly enriched in the proximity of telomere in the tumour samples but not in the non-tumour samples (Fig. 2), suggesting that HBV favours targeting chromosomal elements critical for the maintenance of chromosome stability.

As integration of HBV–DNA into the human genome is considered an early event during hepatocarcinogenesis and can induce chromosomal instability, we next analysed the distribution of HBV breakpoints across individual chromosomes. Although relatively random distribution of integration sites was observed in non-tumour samples, statistically significant enrichment of integration sites on chromosome 5, 16, 17 and 19 was observed in tumour samples (Fig. 2), suggesting that preferential integration exists at the chromosomal level.

### Expression of target genes and effect to HBV integration

The comprehensive HIVID analysis in this large-sample cohort has allowed us to investigate the frequency of recurring tumour-associated integrations in genes. Notably, there were 88 and 17 genes recurrently affected (*n*≥2) by HBV integration in the tumour and non-tumour samples, respectively, which has dramatically expanded our understanding of integration sites of HBV. Apart from the recurrent integrations previously observed in *TERT*, *MLL4* and *CCNE1* genes, novel affected genes (*PTPRD*, *UNC5D*, *NRG3*, *CTNND2* and *AHRR*) were identified, and their expressions were altered at both the transcript and protein levels (Supplementary Figs 6 and 7). Interestingly, these genes have been reported to have oncogenic or tumour suppressive function. In keeping with previously published findings, the expression of some affected genes with oncogenic potential, including *TERT* and *KMT2B*, showed increased expression following HBV integration. Our results suggest that these novel affected genes are, at least in part, functionally relevant to HBV insertion.

**Figure 3:**
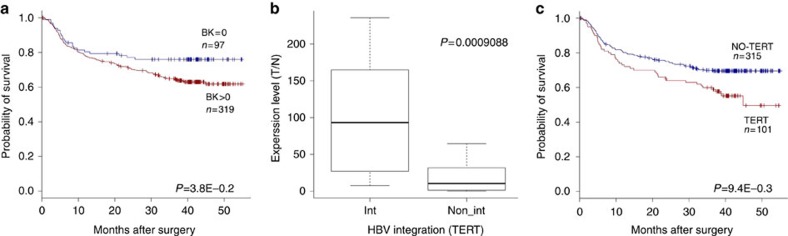
Clinical correlation analysis of HBV integration in HCC. (**a**) Kaplan–Meier survival curves for individuals with (BK>0, *n*=319) versus without (BK=0, *n*=97) HBV integration breakpoints by log-rank test. Those who lacked prognostic information were excluded from the analysis (*n*=10). (**b**) Gene expression levels of *TERT* that frequently harboured HBV integrations in samples with versus without HBV integration events. Gene expression was normalized by the corresponding adjacent, normal control and is represented as the tumour/normal gene expression level. *P* values of unpaired Student's *t* test are shown. In the box plots, the median (50th percentile) is the middle line, with the bottom and top of the box representing the 25th and 75th percentiles of the data, respectively. The ends of the whiskers represent the lowest and highest data within the 1.5 interquartile range (IQR). IQR was defined as the distance between the lower and upper quartiles of the data. (**c**) Kaplan–Meier survival curves for individuals with (*n*=101) versus without (*n*=315) HBV integration in *TERT* by log-rank test.

We noted that individuals with HBV integrations (BK>0) in the tumour displayed a significantly shorter survival than those without HBV integrations (BK=0). Furthermore, HBV integration led to an evident upregulation of *TERT* and patients with HBV integration at *TERT* gene had significantly poorer survival (Fig. 3). Interestingly, tumour-enriched integration into the promoter regions of genes was noted, as compared with the non-tumour genomes (385/3486 versus 57/739; *P*<0.01, *χ*^2^ test). In contrast, the integration breakpoints were preferentially located in the intron region in non-tumour samples (Supplementary Fig. 8). This integration bias indicates that HBV is prone to integrate into promoters in HCC tumours, further affecting transcription of particular genes.

### Breakpoints in HBV genome

**Figure 4:**
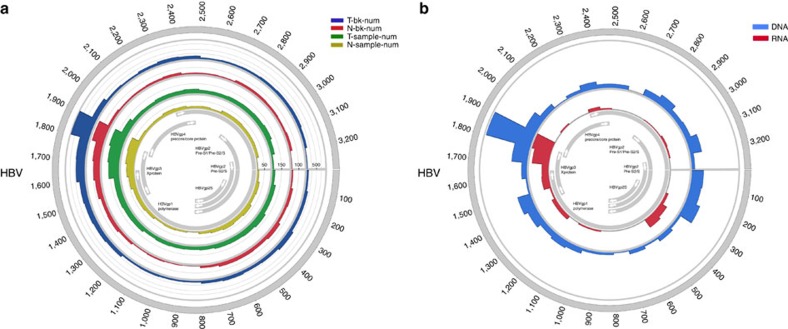
Distribution of integration breakpoints in the HBV genome. (**a**) Distribution of integration breakpoints in the HBV genome in 426 paired samples. Each bar represents the number of HBV integration breakpoints (Tumour: blue, Normal liver tissue: red) or sample frequency (tumour: green, normal liver tissue: yellow) at a particular locus in HBV genome. Histograms were constructed for 100-bp intervals. Histogram axis units represent number of breakpoints, and outer DNA numbering is given in bases. HBV genes with different functions are shown. (**b**) Distribution of integration breakpoints of both DNA and RNA level of 12 paired samples. Histograms were constructed for 100-bp intervals. The number of HBV integration of RNA (red) and DNA (blue) level are shown on the inner and outer circles. Histogram axis units represent number of breakpoints, and outer DNA numbering is given in bases. HBV genes with different functions are shown.

To investigate the integration mechanisms underlying viral–host interactions, we surveyed the breakpoints on the HBV genome. Consistent with previous reports, ∼40% of breakpoints were observed at nucleotides 1,400–1,900 around the 3′-end of the HBx and 5′-end of the Precore/Core genes in both tumour and non-tumour samples(Fig. 4a). To deconvolute HBV integration at the transcriptional level, we randomly selected 12 tumour samples for RNA sequencing and characterized the transcribed viral elements corresponding to nucleotides 1,700–1,900 of HBV genome(Supplementary Table 3). Interestingly, a peak of HBV breakpoints in a region from 300 nt to 500 nt was noted at the transcriptional level, where the S gene was located(Fig. 4b). These viral breakpoints may not only rearrange viral DNA and functional properties but also physically subvert normal control of nearby cellular genes, thus leading to the dysregulation of transcription network in HCC.

Next, we investigated the prevalence of various HBV genotypes across our cohort. The sequence analysis of HBV–DNA from 426 patients with HBV integration revealed the predominant integration of genotype C in 74.9% patients and genotype B in remaining 13.4% patients. The distribution of HBV genotypes in non-HCC liver tissues was similar to tumour tissues. We also noted that the integration rate of B type was significantly higher than C type (Supplementary Fig. 9). No significant correlation between the circulating level of HBV–DNA and the frequency of HBV integration was noted (Supplementary Fig. 10). However, we observed that circulating HBe antigen level had a significant linear correlation with HBV integration in tumour tissues. This suggests that the viral replication capacity could closely influence the HBV integration in human genome (Supplementary Fig. 11).

In our analysis, we observed that multiple HBV fragments could integrate into a single site within the human genome. Further analysis demonstrated that, when compared with non-tumour samples, the breakpoints within tumour samples displayed more diversity with regard to the HBV integration fragments. Although most of the breakpoints were integrated by less than 2 types of HBV fragments, tumour tissues carried more breakpoints integrated by more than 2 types of HBV fragments than non-tumour tissues did (Supplementary Fig. 12), suggesting an increased heterogeneity for HBV integration in tumours.

The mechanism of HBV integration has been thought to involve double-strand break repair, linearized viral DNA invasion/end joining and viral replication, but the details are still unclear^18^. Microhomology (MH)-mediated DNA repair pathways, including MH-mediated end joining, fork stalling and template switching and MH-mediated break-induced repair have been proposed to induce genomic rearrangements^19^,^20^ and other viral insertion^21^. In light of these hypotheses, we searched for the MH sequences between the cellular and the inserted HBV DNA near the integration sites, and found they were significantly enriched with the increase of the MH length, suggesting the potential involvement of MH-mediated mechanism (Supplementary Fig. 13), which could be triggered by the genomic instability/fragility near the integration sites (Supplementary Fig. 14).

### Association of HBV integration with sex and clinical outcome

**Figure 5:**
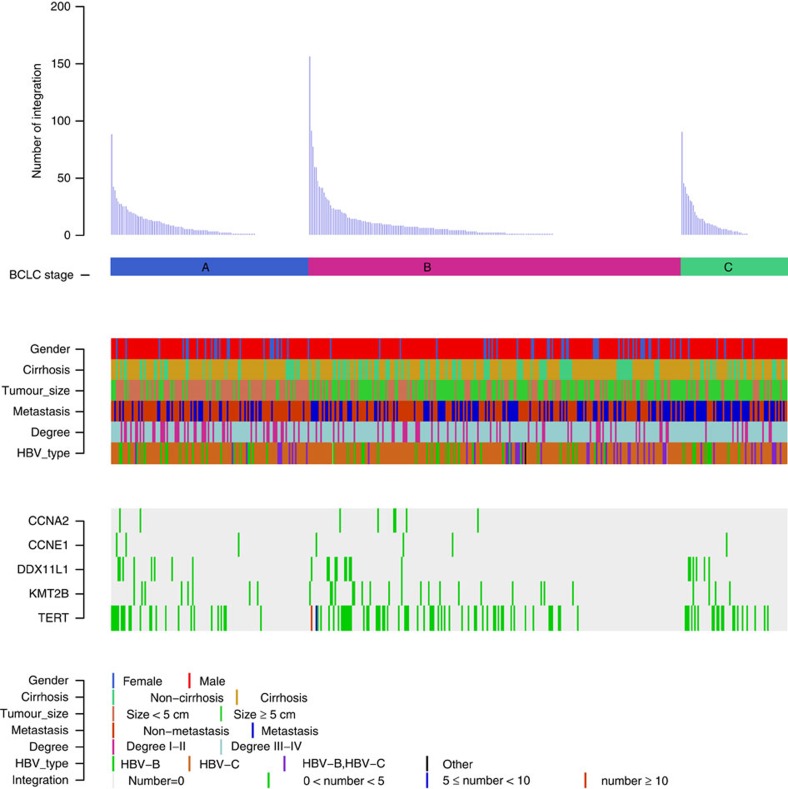
Clinical annotation of HBV integration sites in 426 HCC samples. All panels are aligned with vertical tracks representing 426 individuals. The data are sorted by BCLC stage, gender, cirrhosis, tumour size, metastasis, Edmonson-Steiner classification and HBV type. The bottom heat map shows the distribution of HBV integrations into the five recurrent HBV targeted genes in HCC samples.

**Figure 6:**
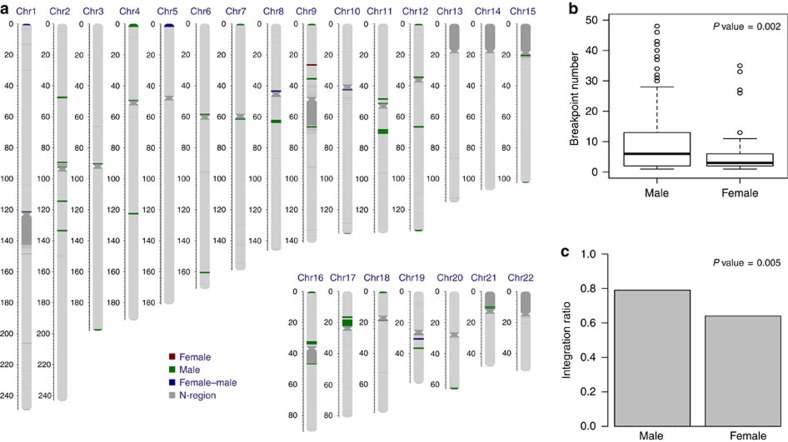
HBV integration preference in male versus female HCC samples. (**a**) Distribution of the enriched HBV integration regions in tumour samples. The red bars indicate the enriched regions found only in female samples, green bars represent enriched regions only found in male samples, and blue bars mean enriched regions found in both. In every 1M window of chromosomal region, the observed number of integration sites was tested against the expected number where all integrations distributed randomly across the genome. Enrichment was defined as *P* value smaller than 0.05 calculated by *χ*^2^ test, which was performed separately in male and female samples. (**b**) Comparison of the numbers of breakpoints in tumour tissues of male and female samples. The HBV breakpoints number of male (left) and female (right) are shown. The box plots show the median (horizontal bar), 25th and 75th percentiles, and the whiskers of the plots show the smallest and largest values. *P* values was calculated by unpaired Student's *t* test. (**c**) Comparison of integration ratio of male and female HCC samples. The HBV integration ratio of male (left) and female (right) HCC samples are shown. *P* values were calculated by *χ*^2^ test.

As HBV integrations may be specific biomarkers for prediction of clinical outcomes, we analysed the association between viral integration and clinicopathological parameters in HCCs (Fig. 5 and Supplementary Table 4). Consistent with a male predominance in HCCs, males had much higher HBV integrations than females in tumour genomes (Supplementary Table 5). Specifically, we observed that substantial genomic regions were preferably integrated by HBV in males in Chromosomes 2 and 17, but not in females (Fig. 6), and HBV integrations in these regions were closely associated with prognosis (Supplementary Fig. 15). Notably, many genes in chromosome 17p are known to play important roles in hepatocarcinogenesis^22^ including the key tumour suppressor *TP53* and master inhibitor of liver regeneration *MKK4* (refs 23, 24, 25). Furthermore, we found integrations in males were prone to locate into the region of core protein (*P*=0.01, *χ*^2^ test) in the HCC genome when compared with females.

### Correlation of HBV integration with Cirrhosis

Clinically, some patients with HBV-related HCC have a lower incidence of cirrhosis, suggesting that critical oncogenes or tumour suppressor genes were involved in hepatocarcinogenesis without inflicting chronic inflammation. Gene annotation revealed a cirrhosis-dependent HBV integration pattern; that is, tumours arising from non-cirrhotic liver displayed a significantly enriched viral integration in the vicinity of putative oncogenes or tumour suppressors such as *KMT2B*, *CCNE1* and *AHRR* compared with those in the cirrhotic liver (Supplementary Data 5 ), suggesting that constitutive activation or inactivation of these genes may contribute to the early onset of HCC without cirrhotic responses.

## Discussion

Here we demonstrate a large scale analysis of HBV–DNA integration sites in liver cancer using HIVID approach. Compared with whole-genome sequencing, HIVID combines HBV fragment capture and massively parallel sequencing, thus dramatically increases the detection efficiency and decreases sequencing complexity and cost. In line with published results on whole-genome sequencing of HBV-associated HCC, HBV integration occurs frequently in both tumour and adjacent non-tumour tissues, with significantly higher insertion rates in tumours^8^,^9^,^10^,^11^. Although integrations of HBV were once thought to be random, our data highlights recurrent integrated regions and greatly expands the list of affected genes. In addition to frequent integrations at *TERT*, *KMT2B* and *CCNE1*, which were known targets of HBV insertion, we discovered massively new recurrent HBV integrations among 426 HCC patients. A significant percentage of them showed expression or function abnormality in tumours and have been shown to be related to cancer progression in other tumour types. Our data provide an important amendment to our understanding of HBV impact on hepatocarcinogenesis, supporting the non-random integration and selection process of HBV targeted genes.

As HBV integration is common and is associated with genomic instability, we examined the distribution of insertion sites in distinct genomic elements^26^,^27^. Not surprisingly, chromosomal fragile sites, known sites of genomic rearrangement in cancer, represent preferred sites for the integration of HBV. In addition, HBV showed an evident preference for CpG islands, in which DNA methylation often precedes the appearance of tumours and are prone to additional chromosomal aberrations, such as loss of heterozygosity. As virus negative tumours have fewer epigenetic and genetic alterations^28^, epigenetic instability via CpG islands methylation/demethylation may contribute to chromosomal instability in HBV-related HCCs. More importantly, the enrichment of HBV integration events was also observed in the proximity of telomere, which plays an important role in maintaining genome stability. Regardless of the mechanism, dysfunction of telomere function can lead to extensive DNA amplification, large terminal deletions and generate many types of rearrangements commonly associated with human cancers^29^.

Although specific sequence features near the fusion breakpoints were not identified in the host genome, there were a handful of hotspots in the tumour genome where multiple HBV fragments could integrate. This is consistent with a non-random integration model followed by a positive selection during hepatocarcinogenesis. Other than HBx proteins surrounding DR1 or DR2 sites, RNA sequencing revealed an enriched expression and favoured integration of preS and S protein in the tumour samples, in accordance with the role of the truncated preS2/S protein in mediating stimulation of cellular genes, in trans^30^.

Despite a significant gender skew of HCC in male, little is known about the difference of HBV integration between genders. Herein, we show that HBV was prone to integrate in male tumour genome and, more importantly, had a chromosome preference. HBV integration into chromosomes2 and 17 was favoured in male tumours, the latter of which contains the *TP53* gene and is frequently lost in the human cancers. In addition to aberrations of *TP53* gene on chromosome 17p13.1, other genes on 17p13.3 may also play a role in hepatocarcinogenesis^22^. Notably, located on 17p adjacent to the *TP53* gene, *MKK4* was recently identified as a master inhibitor of liver regeneration^23^,^24^. As *MKK4* is one of the most consistently mutated genes across tumour types^25^, concurrent inactivation of *TP53* and *MKK4* following HBV integration may constitute an enabling mechanism for genome destabilization and hepatocarcinogenesis. Consistently, patients with HBV integration in chromosomes 2 and 17 displayed significantly poorer survival than in other chromosomes.

Other than inactivating tumour suppressive programs, HBV integrations into different genomic locations can result in a substantial enhancement of distinct oncogenic genes, which may have a profound influence on phenotypic characteristics. In support of this notion, a significant proportion of HBV-associated HCCs develop in the absence of cirrhosis. We identified several important oncogenes preferentially affected by HBV integration in the non-cirrhotic HCC samples, suggesting that constitutive activation or inactivation of these genes may contribute to the early onset of HCC without inflicting cirrhotic responses. Further work is warranted to delineate the complex network of these HBV targeted genes and its overall impact on cellular phenotype.

Collectively, our work provides a large scale and unbiased HBV integration map in HCC, revealing the preference of integration occurring within regions of the genome prone to DNA mutations or rearrangements and novel target genes recurrently affected by HBV integration. Together with the inserted HBV fragments, these characteristics may endow HBV integration a greater opportunity to induce crucial oncogenic alterations to host genes in a gender- and cirrhosis-dependent manner, and eventually leading to HCC development in patients with chronic HBV infection.

## Methods

### Sample and DNA extraction

All samples were obtained from Eastern Hepatobiliary Surgery Hospital, Shanghai, between 2009 and 2010. Tumour tissues and paired adjacent non-tumour tissue were resected from patients undergoing primary hepatectomy by experienced surgeons. Specimens were immediately cryopreserved in −80 °C following resection. All operations were carried out carefully to avoid contamination between samples. DNA was extracted from paired tumour and normal adjacent samples using Genomic DNA Mini Kit (Invitrogen, Life Technologies) according to the manufactory's instruction. All HBV–HCCs, and paired adjacent non-tumour tissue are clinically and pathologically characterized by two independent pathologists and radiologists. Demographic and clinical features of the patients were systematically collected and summarized in Supplementary Table 1. This study was approved by the ethical committee of EHBH hospital and informed consent was obtained from each patient. The inclusion criteria for this study included: (i) HBV-positive HCCs and paired adjacent non-tumour tissues (ii) obtained from consenting patients and (iii) all samples are HCV-negative and HIV-negative and (iv) without for autoimmune hepatitis and metabolic and/or genetics disorders such as Wilson's disease, hemochromatosis.

### RNA extraction and real-time quantitative PCR

Total RNAs were extracted from tumours or paired adjacent non-tumour samples using MagMAX mirVana Total RNA Isolation Kit (Ambion, Life Technologies) according to the manufactory's instruction. The concentration and quality of RNAs were determined by Nanodrop 2000 (Thermo Fisher Scientific). The quantitative PCRs were performed using Power SYBR-Green PCR Master Mix (Life Technologies, Carlsbad, CA, USA), primer pair sets and Applied Biosystems ViiA7 Real-time System (Life Technologies) under the conditions of 10 min at 95 °C followed by 40 cycles at 95 °C for 15 s and at 60 °C for 60 s. Beta actin was used as a housekeeping control. The sequences of the primers are listed in Supplementary Table 2.

### HBV capture experiment

We designed the sequence-capture probes according to eight types of HBV genome (A, B, C, D, E, F, G and H) sequences and these probes were produced by MyGenostics. DNA fragments ∼150–200 bp were obtained after shearing genomic DNA by Covaris E-210 (Covaris, Inc., Woburn, MA). These fragments were purified, end blunted, ‘A' tailed and adaptor ligated. The products of ligation were amplified by PCR and constructed into DNA sequencing library. Bioanalyzer 2100 (Agilent Technologies, Santa Clara, CA ) was used to quantify the concentration of the DNA library. Next, hybridization was performed according to the instruction of Target Enrichment Protocol (GenCapTM Enrichment, MyGenostics, USA). This process was carried out at 65 °C for 24 h and the un-targeted fragments were then removed by washing buffer. These eluted fragments were amplified by PCR and were further processed to Paired end 100-bp read-length for sequencing by the HiSeq 2000 sequencer (Illumina Inc., San Diego, CA).

### Breakpoints detection and annotation of HBV integration sites

HIVID pipeline was used for the breakpoints detection^17^. First, clean reads were obtained through removing low quality and duplicated reads, as well as adaptor-contaminated reads. Burrows-Wheeler Aligner (BWA) was used to align clean reads onto human (NCBI build 37, HG19) and HBV genome (AF090842.1, AB033554.1, AB014381.1, M32138.1, AB032431.1, AB036910.1, AB064310 and AY090454.1). The paired end reads that can be mapped to human or HBV reference genome with both ends were removed. The remained reads were paired-end assembled to reconstruct fragment sequences, which was to locate the position of breakpoints more precisely. Subsequently, reference mapping was performed again to re-map the paired-end-assembled reads onto human and HBV genome using BWA (ref. 31). The position of a breakpoint was defined as the junction of human and HBV sequence in a paired-end-assembled read. To minimize the impact of different sequencing data amount of each sample, the normalized support-reads number (Norm value) was introduced, which was equal to the supported reads number of each HBV breakpoint per million clean read pairs. The breakpoints with Norm value ≥2 were retained. The Sanger sequencing validation rate for the selected breakpoints was 81.1%. ANNOVAR was used to do the annotation for the integrated breakpoints^17^,^31^.

### Process of detecting the integration breakpoints by RNA sequencing

We randomly selected 12 tumour samples undergoing HIVID analysis to perform RNA sequencing. Total RNA isolated with TRIzol reagent was treated with RNase-free DNaseI (New England BioLabs) at 37 °C for 10  min. The Dynabeads mRNA Purification Kit (Life Technologies) was used to isolate mRNA from the total RNA samples. RNA-seq libraries were sequenced as paired-end 100 bp sequence tags using the standard Solexa pipeline. Integration sites were analysed by using the transcriptome data according to previously described method^17^. We removed reads that perfectly aligned to human or HBV genome and reserved chimeric paired-end reads. These chimeric reads were composed of both human genome sequence and HBV genome sequence, and were used to identify HBV integration breakpoints in the transcriptome.

### PCR and sanger sequencing validation

PCR and Sanger sequencing were used to verify the selected HBV integration breakpoints from HIVID. All the samples used for SANGER validation are left-over samples from previous rounds of the capture approach. PCR primers were designed based on the paired-end assembled fragment, in which one primer located in human genome and the other in HBV genome. PCR were performed by GeneAmp PCR System 9700 thermal cycler and then preceded to Sanger sequencing on Applied Biosystems 3730 × DNA analyzer (Life Technologies, Inc.).

### Immunohistochemical staining

Formalin fixed and paraffin-embedded sections (4 μm) were subjected to immunohistochemical staining. The slides were incubated overnight at 4 °C with primary antibodies, including rabbit anti-*AHRR* (1:100, ab108518, Abcam), rabbit anti-*PTPRD*(1:100, LS-B9625, LifeSpan Biosciences), rabbit anti-*NRG3*(1:200, ab83704, Abcam) and mouse anti-*UNC5D* (1:100, ab58141, Abcam). Anti-rabbit or anti-mouse horseradish peroxidase-conjugated secondary antibodies (Santa Cruz Biotechnology, Santa Cruz, CA) were applied. Finally, diaminobenzidine colorimetric reagent solution from Dako (Carpinteria, CA) was used and followed by hematoxylin counterstaining (Sigma Chemical Co). Tissue slides were scanned with an Aperio ScanScope GL, and the Aperio ImageScope software (Aperio Technologies, Vista,CA) was used to assess the scanned images based on the percentage of positively stained cells and staining intensity. Expression levels of these proteins in all clinical samples were quantified.

### Data availability

DNA sequencing data and RNA-seq data that support this study have been deposited in the Sequence Read Archive (SRA) database under the accession codes SRA335342 and SRA447498, respectively. The authors declare that all other data is present in the Article and its Supplementary Information Files or available from the authors upon request.

## Additional information

**How to cite this article:** Zhao, L-H. *et al*. Genomic and oncogenic preference of HBV integration in hepatocellular carcinoma. *Nat. Commun.* **7**, 12992 doi: 10.1038/ncomms12992 (2016).

## Supplementary Material

Supplementary Information Supplementary Figures 1-15 and Supplementary Tables 1-5.

Supplementary Data 1 Sanger validation.

Supplementary Data 2 Breakpoints in Tumor Samples.

Supplementary Data 3 Breakpoints in Normal Samples.

Supplementary Data 4 Integration Frequency in Tumor and Normal Samples.

Supplementary Data 5 Integration Frequency in cirrhotic(CRS) and non-cirrhotic(no-CRS) Tumor Samples.

## References

[b1] Forner A., Llovet J. M. & Bruix J. Hepatocellular carcinoma. Lancet 379, 1245–1255 (2012). 22353262 10.1016/S0140-6736(11)61347-0 PMC13537732

[b2] Siegel R., Naishadham D. & Jemal A. Cancer statistics, 2013. CA Cancer J. Clin. 63, 11–30 (2013). 23335087 10.3322/caac.21166

[b3] Chen J. G. & Zhang S. W. Liver cancer epidemic in China: past, present and future. Semin. Cancer Biol. 21, 59–69 (2011). 21144900 10.1016/j.semcancer.2010.11.002

[b4] Shafritz D. A. . Integration of hepatitis B virus DNA into the genome of liver cells in chronic liver disease and hepatocellular carcinoma. Studies in percutaneous liver biopsies and post-mortem tissue specimens. N. Engl. J. Med. 305, 1067–1073 (1981). 6268980 10.1056/NEJM198110293051807

[b5] Hanahan D. & Weinberg R. A. Hallmarks of cancer: the next generation. Cell 144, 646–674 (2011). 21376230 10.1016/j.cell.2011.02.013

[b6] Lau C. C. . Viral-human chimeric transcript predisposes risk to liver cancer development and progression. Cancer Cell 25, 335–349 (2014). 24582836 10.1016/j.ccr.2014.01.030

[b7] Matsubara K. & Tokino T. Integration of hepatitis B virus DNA and its implications for hepatocarcinogenesis. Mol. Biol. Med. 7, 243–260 (1990). 2170810

[b8] Sung W. K. . Genome-wide survey of recurrent HBV integration in hepatocellular carcinoma. Nat. Genet. 44, 765–769 (2012). 22634754 10.1038/ng.2295

[b9] Ding D. . Recurrent targeted genes of hepatitis B virus in the liver cancer genomes identified by a next-generation sequencing-based approach. PLoS Genet. 8, e1003065 (2012). 23236287 10.1371/journal.pgen.1003065 PMC3516541

[b10] Li X. . The function of targeted host genes determines the oncogenicity of HBV integration in hepatocellular carcinoma. J. Hepatol. 60, 975–984 (2014). 24362074 10.1016/j.jhep.2013.12.014

[b11] Jiang Z. . The effects of hepatitis B virus integration into the genomes of hepatocellular carcinoma patients. Genome Res. 22, 593–601 (2012). 22267523 10.1101/gr.133926.111 PMC3317142

[b12] Yuen M. F., Hou J. L. & Chutaputti A. Hepatocellular carcinoma in the Asia pacific region. J. Gastroenterol. Hepatol. 24, 346–353 (2009). 19220670 10.1111/j.1440-1746.2009.05784.x

[b13] Chu C. M. . Basal core promoter mutation is associated with progression to cirrhosis rather than hepatocellular carcinoma in chronic hepatitis B virus infection. Br. J. Cancer 107, 2010–2015 (2012). 23079574 10.1038/bjc.2012.474 PMC3516680

[b14] Perz J. F. . The contributions of hepatitis B virus and hepatitis C virus infections to cirrhosis and primary liver cancer worldwide. J. Hepatol. 45, 529–538 (2006). 16879891 10.1016/j.jhep.2006.05.013

[b15] Chu C. M. . Sex difference in chronic hepatitis B virus infection: an appraisal based on the status of hepatitis B e antigen and antibody. Hepatology 3, 947–950 (1983). 6313507 10.1002/hep.1840030611

[b16] Huang Y. T. . Lifetime risk and sex difference of hepatocellular carcinoma among patients with chronic hepatitis B and C. J. Clin. Oncol. 29, 3643–3650 (2011). 21859997 10.1200/JCO.2011.36.2335 PMC4874144

[b17] Li W. . HIVID: an efficient method to detect HBV integration using low coverage sequencing. Genomics 102, 338–344 (2013). 23867110 10.1016/j.ygeno.2013.07.002

[b18] Hino O., Ohtake K. & Rogler C. E. Features of two hepatitis B virus (HBV) DNA integrations suggest mechanisms of HBV integration. J. Virol. 63, 2638–2643 (1989). 2542576 10.1128/jvi.63.6.2638-2643.1989 PMC250746

[b19] Verdin H. . Microhomology-mediated mechanisms underlie non-recurrent disease-causing microdeletions of the FOXL2 gene or its regulatory domain. PLoS Genet. 9, e1003358 (2013). 23516377 10.1371/journal.pgen.1003358 PMC3597517

[b20] Liu P. . Mechanisms for recurrent and complex human genomic rearrangements. Curr. Opin. Genet. Dev. 22, 211–220 (2012). 22440479 10.1016/j.gde.2012.02.012 PMC3378805

[b21] Hu Z. . Genome-wide profiling of HPV integration in cervical cancer identifies clustered genomic hot spots and a potential microhomology-mediated integration mechanism. Nat. Genet. 47, 158–163 (2015). 25581428 10.1038/ng.3178

[b22] Zhao X. . The minimum LOH region defined on chromosome 17p13.3 in human hepatocellular carcinoma with gene content analysis. Cancer Lett. 190, 221–232 (2003). 12565177 10.1016/s0304-3835(02)00622-5

[b23] Yeasmin S. . MKK4 acts as a potential tumor suppressor in ovarian cancer. Tumour Biol. 32, 661–670 (2011). 21487811 10.1007/s13277-011-0166-5

[b24] Wuestefeld T. . A Direct *in vivo* RNAi screen identifies MKK4 as a key regulator of liver regeneration. Cell 153, 389–401 (2013). 23582328 10.1016/j.cell.2013.03.026

[b25] Cunningham S. C. . Targeted deletion of MKK4 in cancer cells: a detrimental phenotype manifests as decreased experimental metastasis and suggests a counterweight to the evolution of tumor-suppressor loss. Cancer Res. 66, 5560–5564 (2006). 16740690 10.1158/0008-5472.CAN-06-0555

[b26] Feitelson M. A. & Lee J. Hepatitis B virus integration, fragile sites, and hepatocarcinogenesis. Cancer Lett. 252, 157–170 (2007). 17188425 10.1016/j.canlet.2006.11.010

[b27] Matsuzaki Y. . HBV genome integration and genetic instability in HBsAg-negative and anti-HCV-positive hepatocellular carcinoma in Japan. Cancer Lett. 119, 53–61 (1997). 18372522 10.1016/s0304-3835(97)00249-8

[b28] Katoh H. . Epigenetic instability and chromosomal instability in hepatocellular carcinoma. Am. J. Pathol. 168, 1375–1384 (2006). 16565510 10.2353/ajpath.2006.050989 PMC2216681

[b29] Bailey S. M. & Murnane J. P. Telomeres, chromosome instability and cancer. Nucleic Acids Res. 34, 2408–2417 (2006). 16682448 10.1093/nar/gkl303 PMC1458522

[b30] Kekule A. S. . The preS2/S region of integrated hepatitis B virus DNA encodes a transcriptional transactivator. Nature 343, 457–461 (1990). 2153938 10.1038/343457a0

[b31] Li H. & Durbin R. Fast and accurate short read alignment with Burrows-Wheeler transform. Bioinformatics 25, 1754–1760 (2009). 19451168 10.1093/bioinformatics/btp324 PMC2705234

