# Erratum: Genomic and oncogenic preference of HBV integration in hepatocellular carcinoma

**DOI:** 10.1038/ncomms13591

**Published:** 2016-11-08

**Authors:** Ling-Hao Zhao, Xiao Liu, He-Xin Yan, Wei-Yang Li, Xi Zeng, Yuan Yang, Jie Zhao, Shi-Ping Liu, Xue-Han Zhuang, Chuan Lin, Chen-Jie Qin, Yi Zhao, Ze-Ya Pan, Gang Huang, Hui Liu, Jin Zhang, Ruo-Yu Wang, Yun Yang, Wen Wen, Gui-Shuai Lv, Hui-Lu Zhang, Han Wu, Shuai Huang, Ming-Da Wang, Liang Tang, Hong-Zhi Cao, Ling Wang, Tin-Lap Lee, Hui Jiang, Ye-Xiong Tan, Sheng-Xian Yuan, Guo-Jun Hou, Qi-Fei Tao, Qin-Guo Xu, Xiu-Qing Zhang, Meng-Chao Wu, Xun Xu, Jun Wang, Huan-Ming Yang, Wei-Ping Zhou, Hong-Yang Wang

Nature Communications **7**: Article number: 12992; DOI: 10.1038/ncomms12992 (2016); Published: 10 05 2016; Updated: 11 08 2016

The original version of this Article contained an error in the spelling of the author Tin-Lap Lee, which was incorrectly given as T.P. Lee. This has now been corrected in both the PDF and HTML versions of the Article.

