# Supplementary material for: Genomic and oncogenic preference of HBV integration in hepatocellular carcinoma
Source: Nat Commun. 2016 Oct 5;7:12992. doi: 10.1038/ncomms12992 (PMC5059470; doi:10.1038/ncomms12992)
Supplement: Supplementary Information — Supplementary Figures 1-15 and Supplementary Tables 1-5. [file ncomms12992-s1.pdf]

Supplementary Information

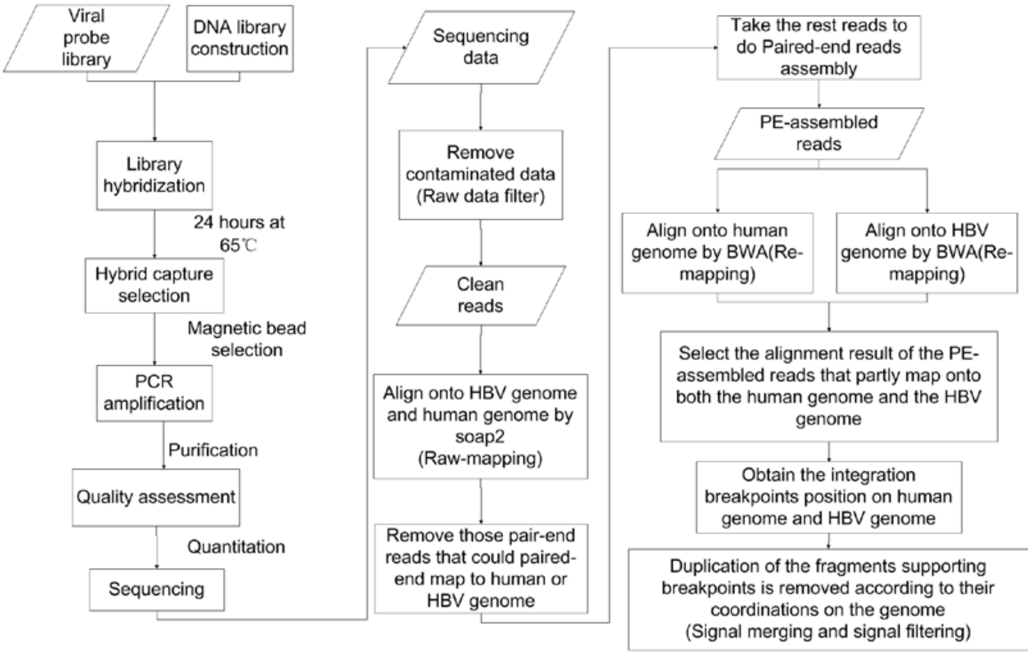

Supplementary Figure. 1 The pipeline of workflow. The pipeline includes the workflow of experiment and bioinformatics process. In the process, we performed Raw-mapping with filtered raw data and assembled the chimeric paired-end reads. Paired-endassembled reads wereconducted to go through Re-mapping to locate the HBV integrations sites. Signal merging and signal filter were performed to obtain the final results.

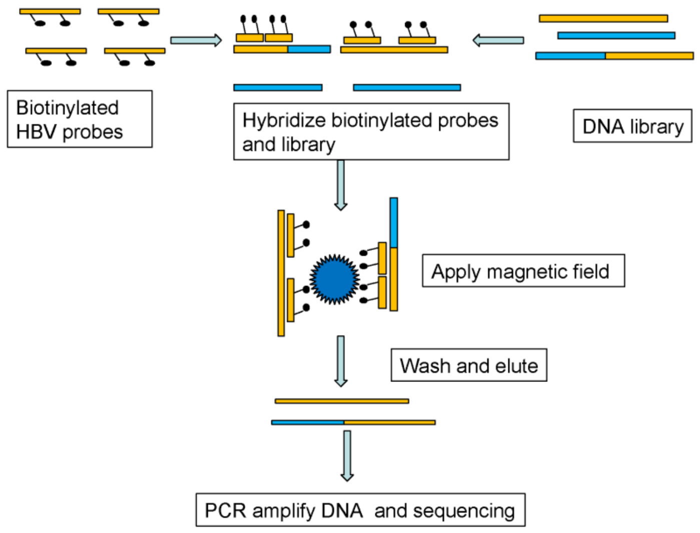

Supplementary Figure. 2 HBV capture workflow in HIVID. The yellow colored represented the HBV fragments, and the blue colored represented the human genome fragments. DNA libraries were hybridized with HBV probes at 65 °C for 24 hours and then washed to remove un-captured fragments.The eluted fragments were amplified by 16 cycles of PCR to generate libraries for sequencing.

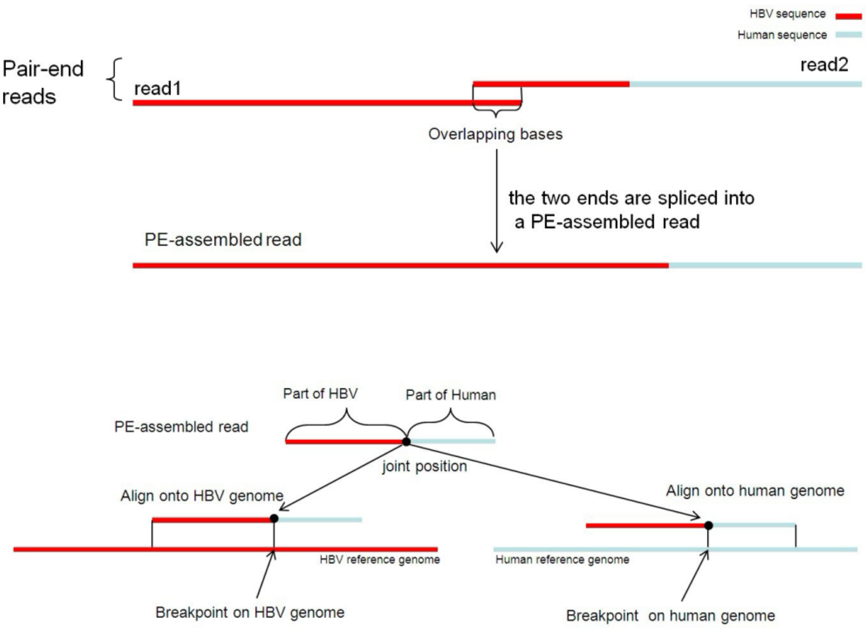

Supplementary Figure. 3 Insight of computational process in HIVID. (a) Principle of pair-end reads assembly. The tail of an upstream end (read1) and the head of a downstream end (read2) were spliced into one continuous sequence 10bp (b) Determination of breakpoint. A PE-assembled read consists of the part of human sequence and part of HBV sequence was depicted. The joint position was the breakpoint for HBV integration.

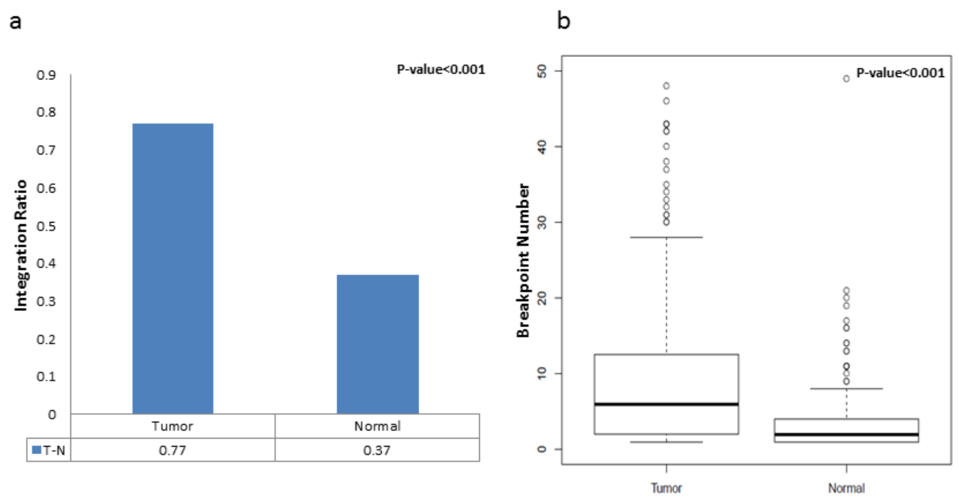

Supplementary Figure. 4 Comparison of integration ratio and number of breakpoints in tumor versus non-tumor tissues in 426 paired samples. The integration ratio (a) and number of breakpoints (b) in tumor tissues compared to non-tumor tissues. (a) P values were calculated by Chi-squared test. (b) P values were calculated by unpaired Student's t test. In the box plots, the median (50th percentile) is the black middle line, with the bottom and top of the box representing the 25th(Q1) and 75th(Q3) percentiles of the data, respectively. The whiskers are the upper and lower adjacent values within Q3+1.5(Q3−Q1) and Q1−1.5(Q3−Q1). The hollow Points represent outliers.

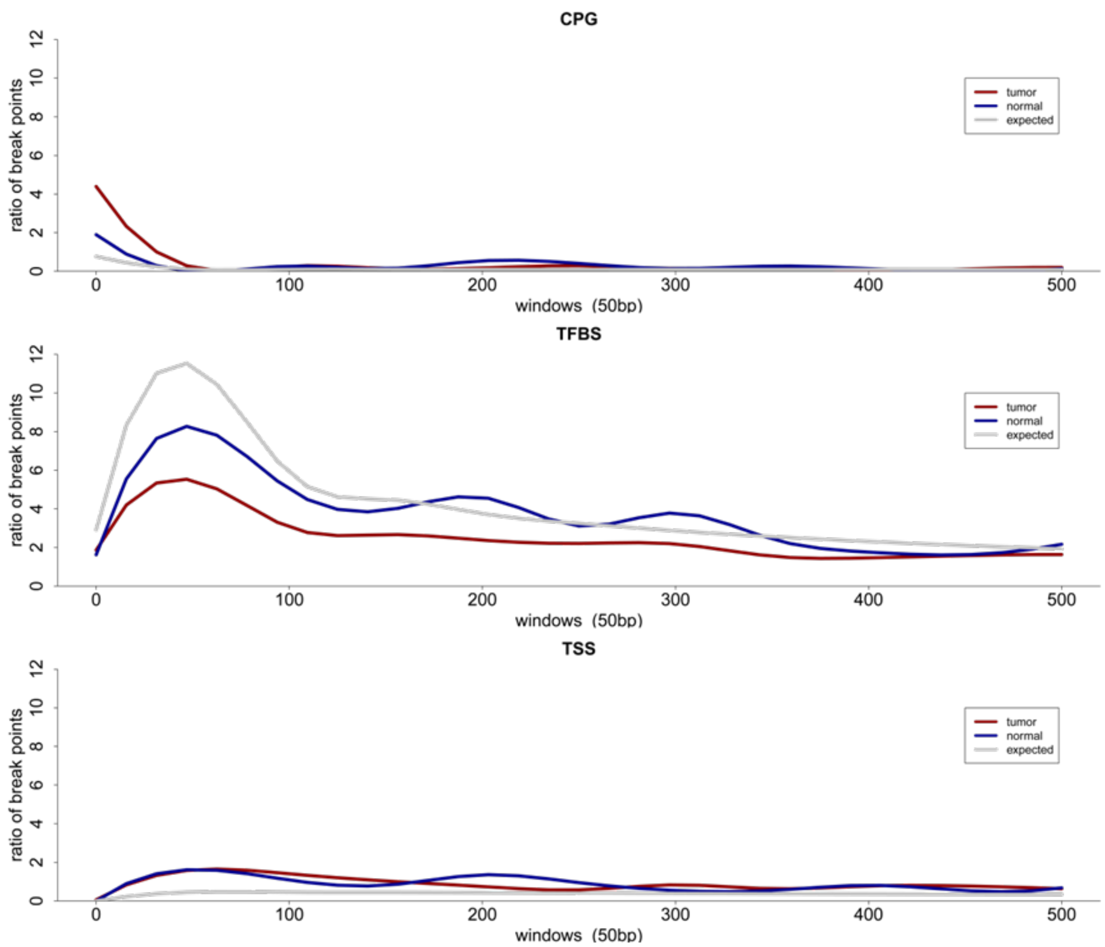

Supplementary Figure. 5 Distribution of integration breakpoints in CPG, TFBS, TSS. The number of integrated breakpoints in CpG islands, transcription factor binding sites(TFBS) and transcription start sites(TSS) were calculated. A uniformly random distribution of breakpoints across the entire human genome was used to calculate the expected number. Gray line, expected percentage of HBV-integrated breakpoints. Blue line, the observed percentage of HBV-integrated breakpoints in non-tumor tissues. Red line, the observed percentage of HBV-integrated breakpoints in tumor tissues.

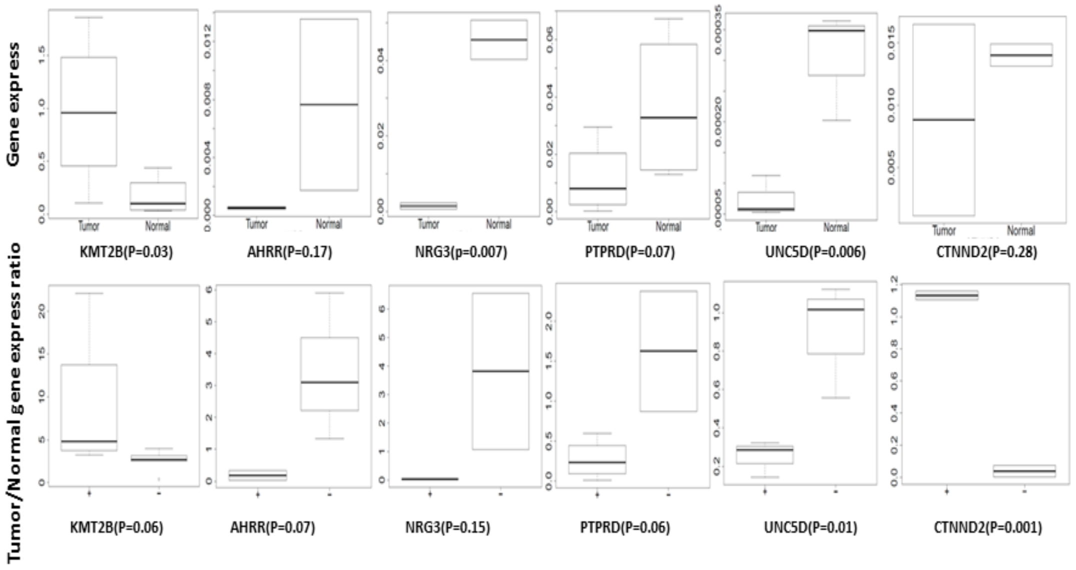

Supplementary Figure. 6 Effects of HBV integration on gene expression. (a) RNA expression from genes that frequently harbored HBV integrations in tumor tissues versus adjacent non-tumor tissues. The number of paired samples surveyed in KMT2B(n=15), AHRR(n=3), NRG3(n=2), PTPRD(n=3), UNC5D(n=3) and CTNND2(n=2) and their associated P values are calculated by paired Student's t test are shown (b) Comparison of gene expression levels in tumor from genes that frequently underwent integration events in samples with or without HBV integration. (+) represents samples with integration events; (-) represents sample without integration events. P values were calculated by unpaired Student's t test. In the box plots, the median (50th percentile) is the black middle line, with the bottom and top of the box representing the 25th(Q1) and 75th(Q3) percentiles of the data, respectively.

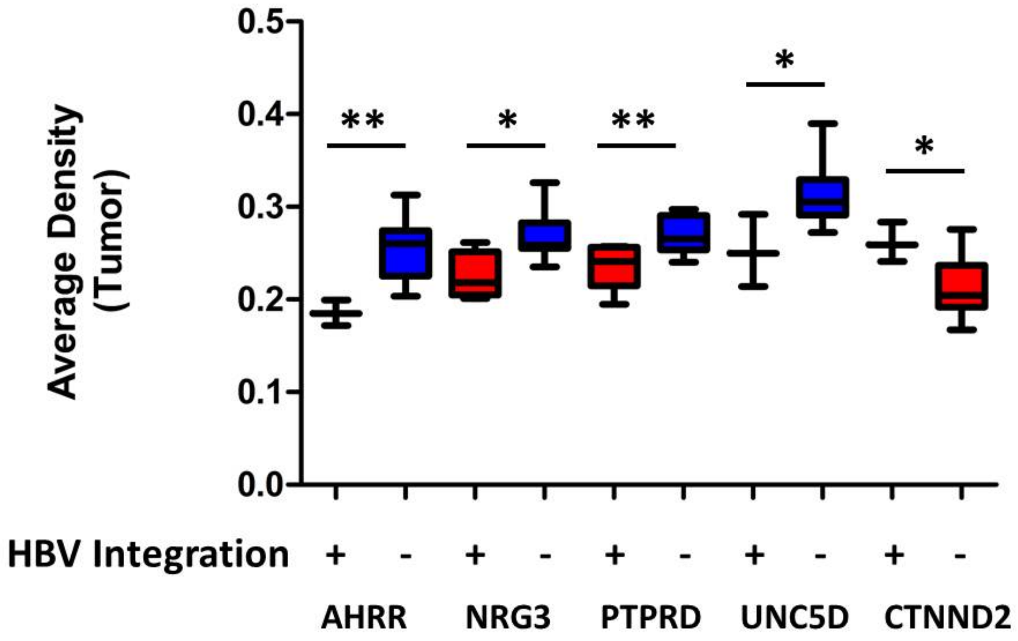

Supplementary Figure.7 Effects of HBV integration on protein expression in HCC samples Relative IHC staining of AHRR, NRG3, PTPRD, UNC5D and CTNND2 in HCC samples with(red) versus without(blue) HBV integration. P values of unpaired Student's t test are shown. In the box plots, the median (50th percentile) is the middle line, with the bottom and top of the box representing the 25th and 75th percentiles of the data, respectively. The ends of the whiskers represent the lowest and highest data within the 1.5 interquartile range (IQR). IQR was defined as the distance between the lower and upper quartiles of the data. * $p<0.05$, **$p<0.005$. P values were calculated by unpaired Student's t test.

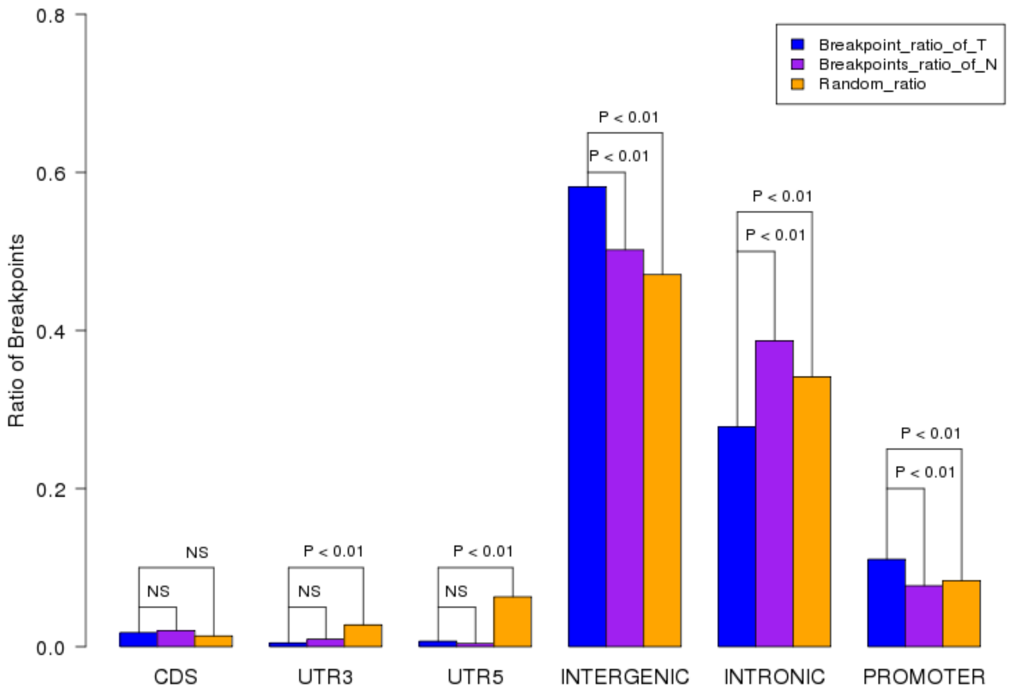

Supplementary Figure.8 Distribution of integration breakpoints in human genetic elements. The number of integrated breakpoints in each genome element was calculated. A uniformly random distribution of breakpoints across the entire human genome was used to calculate the expected ratio of breakpoint number. Yellow bar shows the expected ratio of HBV-integrated breakpoints. Blue bar shows the observed ratio of HBV-integrated breakpoints in tumor tissues. Purple bar shows the observed ratio of HBV-integrated breakpoints in non-tumor tissues. P values were calculated by Chi-squared test.

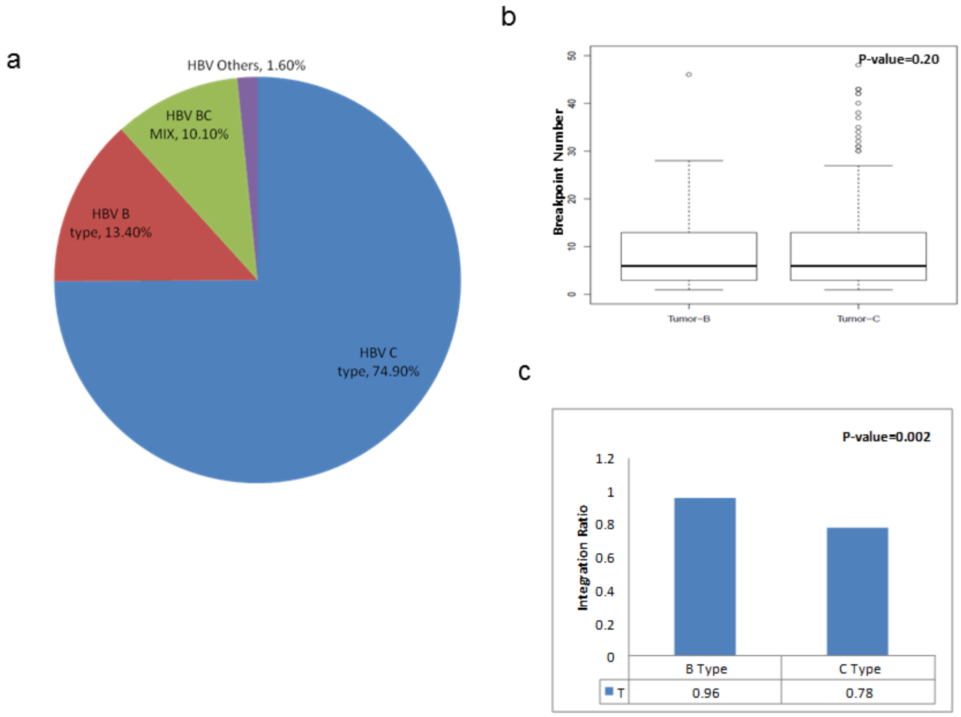

Supplementary Figure.9 Comparison of integration characteristics between HBV genotype B and C (a) HBV genotype proportion in the 426 tumor samples. (b) Comparison of the number of breakpoints between HBV B and C type. In the box plots, the median (50th percentile) is the black middle line, with the bottom and top of the box representing the 25th(Q1) and 75th(Q3) percentiles of the data, respectively. The whiskers are the upper and lower adjacent values within Q3+1.5(Q3−Q1) and Q1−1.5(Q3−Q1). The hollow Points represent outliers. P values were calculated by unpaired Student’s t test. (c) Comparison of integration ratio between HBV genotype B and C. P values were calculated by Chi-square test.

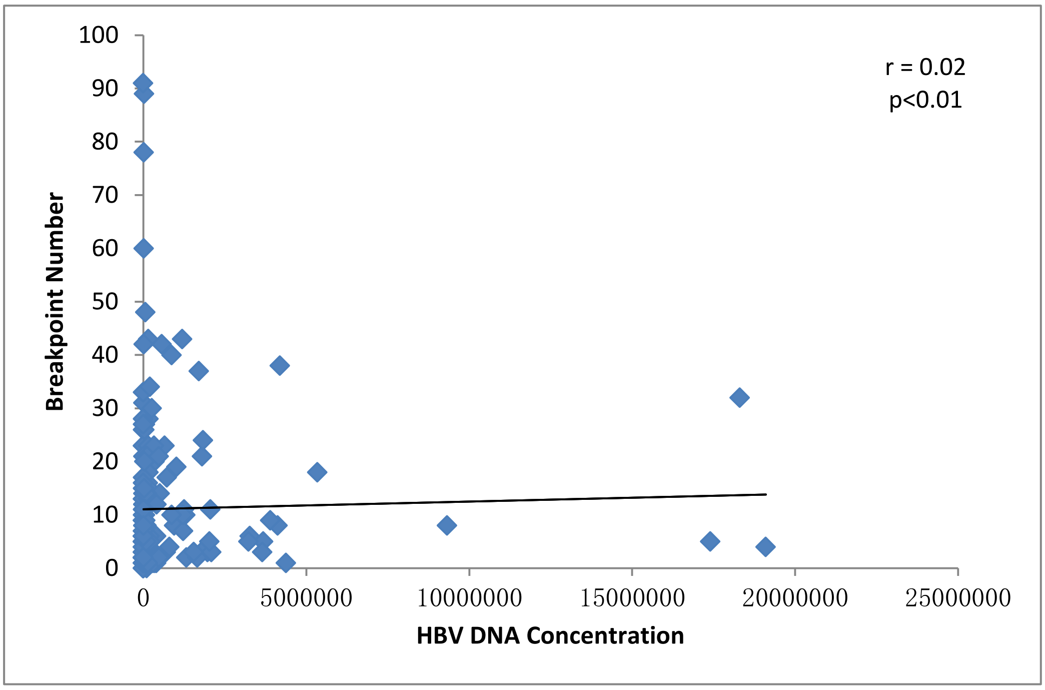

Supplementary Figure.10 Correlation analysis of the circulating level of HBV-DNA with the frequency of HBV integration Breakpoint number of HBV integration and circulating level of HBV DNA in tumor tissues, analyzed by Pearson correlation test ($R = 0.02$; $P < 0.01$).

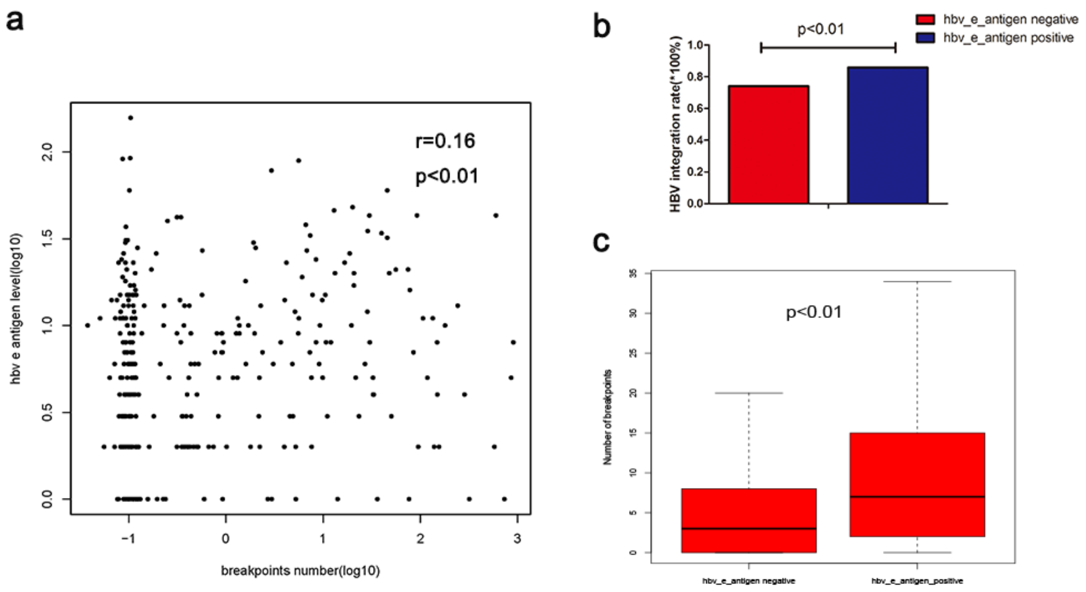

Supplementary Figure.11 Correlation analysis of HBV integration and HBe antigen level in HCC samples (a) Correlation analysis of HBV integration breakpoints with circulating HBe antigen level. (pearson correlation test) (b) Comparison of HBV integration rate between HBe antigen positive VS negative samples, chi-squared test. (c) Comparison of the numbers of breakpoints in tumor tissues of HBe antigen positive and negative samples (p=0.0022). The box plots show the median (horizontal bar), 25th(Q1) and 75th(Q3) percentiles, and the whiskers are the upper and lower adjacent values within Q3+1.5(Q3−Q1) and Q1−1.5(Q3−Q1). P values was calculated by unpaired Student’s t test.

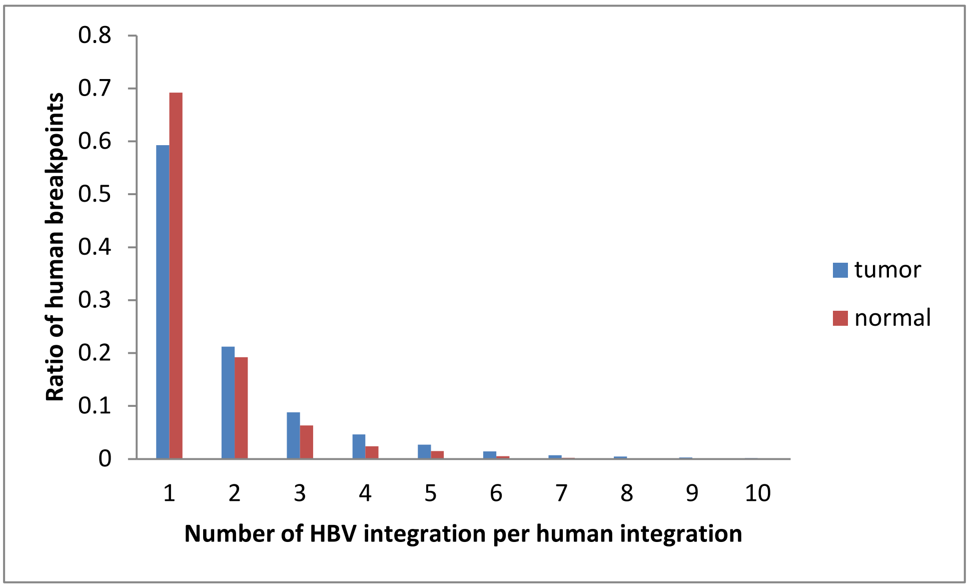

Supplementary Figure. 12 The distribution of breakpoints which have different HBV integration events in tumor and non-tumor samples.

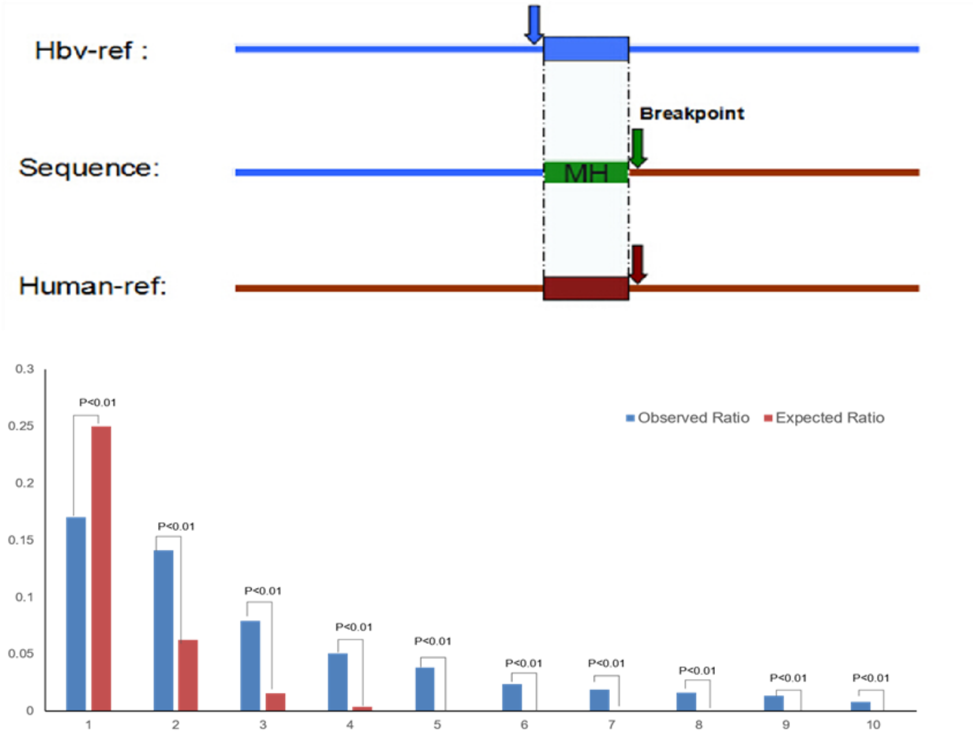

Supplementary Figure. 13 Correlation between MH sequences and the HBV integration events The above is the schematic figure of

homology between human genome and HBV genome flanking the integrated breakpoints. The below is the comparison between observed and expected integration events with different length of homologous sequences. Red bars indicate the expected number while blue bars indicate the observed number. X axis is the length the continuously homologous sequences while Y axis is the percentage of integration events. P values were calculated by Chi-squared test.

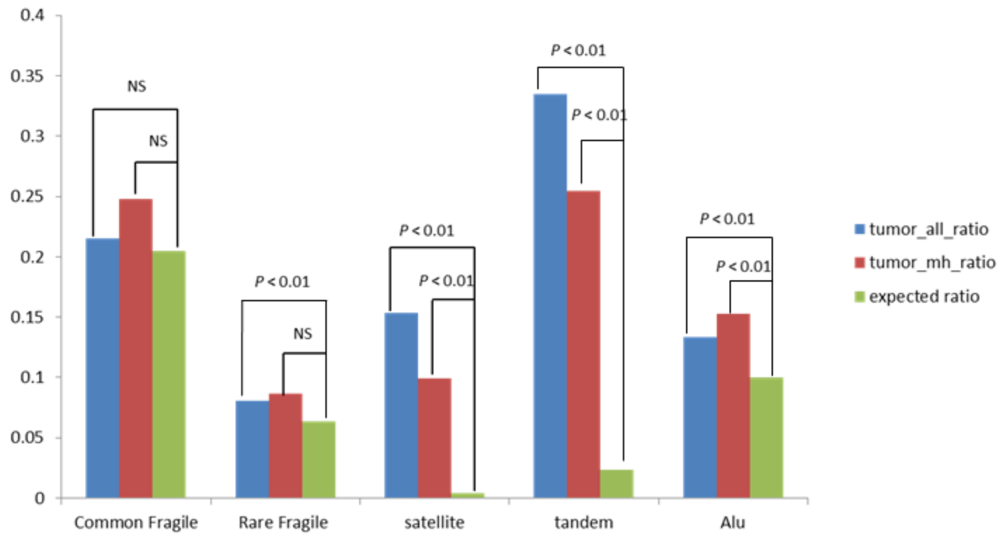

Supplementary Figure. 14 Comparison of ratio of breakpoints in common fragile,rare fragile, satellite, tandem and Alu. X axis represents different elements; Y axis represent ratio of integration breakpoints. Blue bar represents the observed ratio of breakpoints in tumor tissues(tumor_all-ratio); Red bar shows the observed ratio of breakpoints with MH(MH>=3) in tumor tissues(tumor_mh-ratio). Green bar shows the expected ratio of breakpoints(expected ratio). P values

were calculated by Chi-squared test.

.

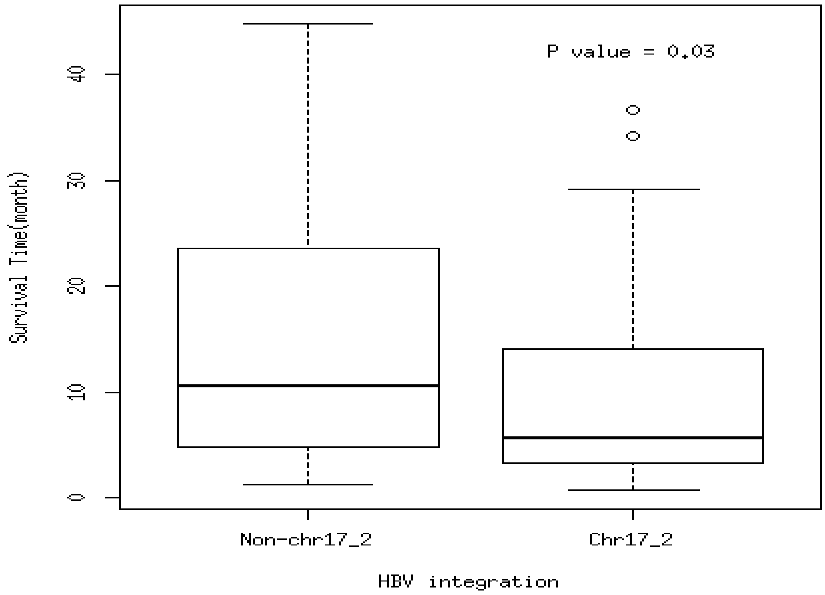

Supplementary Figure. 15 HBV integrations in chromosome 2 and 17 were closely associated with prognosis Comparison of the survival time of patients with or without HBV integration in tumor chromosome 2 and 17. P values were calculated by unpaired Student’s t test. The box plots show the median (middle horizontal bar), 25th(Q1) and 75th(Q3) percentiles, and The whiskers are the upper and lower adjacent values within Q3+1.5(Q3−Q1) and Q1−1.5(Q3−Q1). The hollow Points represent outliers.

Supplementary Table 1: Demographic and clinicopathologic characteristics of 426 HCC patients

Variables	Mean ± SD / n (%)
**Age**, years	52.3 ±10.5
**Sex**	
Female	66 (15.5%)
Male	360 (84.5%)
**HBsAg status**	
positive	426 (100.0%)
**TBIL, μmol/L**	16.9 ±11.4
**ALB, g/L**	42.5 ±4.1
**PT, seconds**	12.1 ±1.0
**AFP, μg/L**	507.3 ±541.9
**Tumor diameter, cm**	6.9 ±4.8
**Edmonson-Steiner classification**	
I- II	99 (23.2%)
III- IV	327 (76.8%)
**Microvascular invasion**	
Absence	241 (56.6%)
Presence	185 (43.4%)
**Satellite nodules**	
No	247 (58.0%)
Yes	179 (42.0%)
**Tumor Capsule**	
Complete	151 (35.4%)
Incomplete	188 (44.1%)
No	87 (20.4%)
**Cirrhosis**	
No	128 (30.0%)
Yes	298 (70.0%)
**Child-Pugh grade**	
A	417 (97.9%)
B	9 (2.1%)

Supplementary Table 2: Sequences of the Primers used for quantitative RT-PCR analysis

Gene	Orientation	Sequence (5' to 3')
TERT	Forward	GAGCTGACGTGGAAGATGAG
	Reverse	CTGACCTCTGCTTCCGACA
KMT2B	Forward	CCAGACCTGCTGCTTGAGTC
	Reverse	GCCAGGAAGATGACAGCAT
AHRR	Forward	CGCCTCAGTGTCAGTTACCT
	Reverse	TGTTCTGGTGCATTACATCC
NRG3	Forward	TACGACGACATATTCCACAGA
	Reverse	GGCTCTTCACCAAGTTCTCTG
β-actin	Forward	AATCGTGCGTGACATTAAGGAG
	Reverse	ACTGTGTTGGCGTACAGGTCTT
PTPRD	Forward	GGTGCACGTAGCCAGGC
	Reverse	CTGTGAGTCTGGTGGATACACTTA
UNC5D	Forward	CATAGAGGAGCCAGATGATGC
	Reverse	TGATGATCAGGTTATGGTCAGC
CTNND2	Forward	GAGCTATGCCTGTTCCAGAC
	Reverse	GTCCACCAGCTCGAGACCT

Supplementary Table 3: Breakpoints in RNA Samples

SAMPLE(RNA-seq)	CHR	Posistion	Left support	Right support	Total support reads
Sample_D081177T	chr8	38955196	0	340	340
Sample_D081177T	chr10	8644547	47	0	47
Sample_D081177T	chr19	36213259	0	5	5
Sample_D080934T	chr20	22732805	97	0	97
Sample_D080696T	chr17	16142730	0	24	24
Sample_D080696T	chr5	166187727	0	321	321
Sample_D080696T	chr18	19507779	76	0	76
Sample_D080696T	chr4	158964287	61	0	61
Sample_D080696T	chr4	158964150	0	48	48
Sample_D080696T	chr4	39928003	0	29	29
Sample_D080696T	chr8	18147699	0	26	26
Sample_D080696T	chr2	100836746	22	0	22
Sample_D080696T	chr20	10746901	0	20	20
Sample_D080696T	chr7	100632468	19	0	19
Sample_D080696T	chr8	110744192	0	13	13
Sample_D080696T	chr8	47368568	0	10	10
Sample_D080696T	chr8	47352554	0	9	9
Sample_D080696T	chr17	25565549	0	6	6
Sample_D079933T	chr3	182607683	0	151	151
Sample_D079933T	chr3	182614486	0	5	5
Sample_D079933T	chr3	182614554	4	0	4
Sample_D079376T	chr19	36200018	65	0	65
Sample_D079376T	chr19	36212377	13	0	13
Sample_D079376T	chr19	36212430	13	0	13
Sample_D079376T	chr19	36212652	0	11	11
Sample_D079376T	chr19	36212712	9	0	9
Sample_D079376T	chr19	36196215	7	0	7
Sample_D078621T	chr3	182061415	3	0	3
Sample_D078621T	chr3	7294807	0	3	3
Sample_D078621T	chr3	182042084	2	0	2
Sample_D078216T	chr11	68051968	0	22	22
Sample_D078216T	chr11	123717339	16	0	16
Sample_D078216T	chr11	123713335	10	0	10
Sample_D078165T	chr5	512163	91	0	91
Sample_D078165T	chr5	512044	46	0	46
Sample_D078165T	chr15	55269070	0	30	30
Sample_D077748T	chr5	1296061	0	319	319
Sample_D077748T	chr5	1296031	6	0	6
Sample_D077450T	chr12	20264192	426	0	426
Sample_D077450T	chr4	189654924	8	0	8
Sample_D077331T	chr22	35501134	0	2357	2357
Sample_D071248T	chr8	48706970	0	11	11

Supplementary Table 4: Integration Ratio in different groups

Variable	Group of patients	Patient number of Integration	Patient number of No-Integration	Integrated ratio	P-value	P-value adjust by multi test(FDR)
**Age**	>50	169	64	0.73	**0.023**	0.1
	<=50	159	34	0.82		
**Gender**	male	286	74	0.79	**0.006**	**0.04**
	female	42	24	0.64		
**Cirrhosis**	no-cirrhosis	102	26	0.8	0.453	0.78
	cirrhosis	226	72	0.76		
**Degree**	1--2	75	25	0.75	0.681	0.78
	3--4	253	73	0.78		
**Tumor Size**	>=5	170	56	0.75	0.361	0.78
	<5	158	42	0.79		
**Involucrum**	none	67	20	0.77	1	1
	intact	116	34	0.77		
**Metastasis**	Yes	140	39	0.78	0.639	0.78
	No	188	59	0.76		
**Microvascular thrombosis**	Yes	145	40	0.78	0.546	0.78
	No	183	58	0.76		
**Barcelona clinical grouping**	A	101	23	0.81	0.3634	0.78
	B	171	64	0.73		
	C	55	11	0.83		
**HBV type**	B	55	2	0.96	**0.001**	**0.01**
	C	251	68	0.79		
**Albumin**	<=42.4	163	51	0.76	0.728	0.78
	>42.4	164	47	0.78		
**Total bilirubin**	<=14.9	166	47	0.78	0.632	0.78
	>14.9	161	51	0.76		
**Time of thrombinogen**	<=12	174	43	0.8	0.115	0.4
	>12	153	55	0.74		
**AFP**	<168.6	165	47	0.78	0.716	0.78
	>168.6	161	51	0.76		

Supplementary Table. 5 Number of enriched regions of HBV integration in genome of male and female patients.

	male	female	male+female
1M	11.33±3.50	3.33±1.03	3.67±1.03
2M	11.67±2.88	5±1.67	5±1.67
4M	11.5±3.14	2.83±1.72	4.17±1.72

We randomly chose 66 of the 360 male samples 6 times and then compared with female samples respectively on 3 different genomic window size [1M , 2M and 4M] for each random sample selection. The ratio of breakpoints on each window was tested against the random distribution. Enriched regions are defined where P values were smaller than 0.05, which was calculated by Chi-Squared tests. Afterwards, we calculated the mean value of numbers of enriched regions and their standard deviations. “male” column indicates the enriched regions found only in female samples, “female” column represents enriched regions only found in male samples, and “male+female” means enriched regions found in both.
